# Lactate and Lactylation: Dual Regulators of T-Cell-Mediated Tumor Immunity and Immunotherapy

**DOI:** 10.3390/biom14121646

**Published:** 2024-12-21

**Authors:** Zhi-Nan Hao, Xiao-Ping Tan, Qing Zhang, Jie Li, Ruohan Xia, Zhaowu Ma

**Affiliations:** 1 Department of Gastroenterology, First Affiliated Hospital of Yangtze University, Health Science Center, Yangtze University, Jingzhou 434023, China; 2023711032@yangtzeu.edu.cn (Z.-N.H.); www95@126.com (Q.Z.); tanxiaohua2222@163.com (J.L.); 2 Digestive Disease Research Institution of Yangtze University, Yangtze University, Jingzhou 434023, China; tanxp1971@163.com; 3 The Third Clinical Medical College of Yangtze University, Jingzhou Hospital of Traditional Chinese Medicine, Jingzhou 434023, China; 4 School of Basic Medicine, Health Science Center, Yangtze University, Jingzhou 434023, China

**Keywords:** lactate, lactylation, T cell, immune modulation, glycolysis, tumor microenvironment

## Abstract

Lactate and its derivative, lactylation, play pivotal roles in modulating immune responses within the tumor microenvironment (TME), particularly in T-cell-mediated cancer immunotherapy. Elevated lactate levels, a hallmark of the Warburg effect, contribute to immune suppression through CD8^+^ T cell functionality and by promoting regulatory T cell (Treg) activity. Lactylation, a post-translational modification (PTM), alters histone and non-histone proteins, influencing gene expression and further reinforcing immune suppression. In the complex TME, lactate and its derivative, lactylation, are not only associated with immune suppression but can also, under certain conditions, exert immunostimulatory effects that enhance cytotoxic responses. This review describes the dual roles of lactate and lactylation in T-cell-mediated tumor immunity, analyzing how these factors contribute to immune evasion, therapeutic resistance, and immune activation. Furthermore, the article highlights emerging therapeutic strategies aimed at inhibiting lactate production or disrupting lactylation pathways to achieve a balanced regulation of these dual effects. These strategies offer new insights into overcoming tumor-induced immune suppression and hold the potential to improve the efficacy of cancer immunotherapies.

## 1. Introduction

T cells are typically categorized into distinct subtypes, with CD8^+^ and CD4^+^ T cells representing the primary classifications. CD4^+^ T cells are further classified into T helper 1 (Th1), Th17, and Tregs. CD8^+^ T cells, also referred to as cytotoxic T lymphocytes (CTLs), play a crucial role in the direct destruction of target cells. Upon the recognition of antigens presented by MHC class I molecules through their T cell receptors (TCRs), CD8^+^ T cells secrete cytotoxic substances, including perforin and granzymes. These molecules either disrupt the structural integrity of the target cell membrane or initiate apoptotic pathways. This mechanism is essential for the facilitation of tumor immunity [1]. Correspondingly, CD4^+^ T cells predominantly modulate the activity of other immune cells via the secretion of cytokines. Th1 cells produce interferon-γ (IFN-γ) and interleukin-2 (IL-2), which play crucial roles in activating macrophages and augmenting the cytotoxic capabilities of T cells. This process is integral to the immune system’s responses against viral infections and tumorigenesis [2]. Th17 cells, through IL-17 secretion, contribute to chronic inflammatory responses and are essential in the development of autoimmune diseases [3]. Treg cells, however, inhibit immune responses by secreting IL-10 and TGF-β, as well as through direct interactions with effector T cells. This activity is essential for mitigating [4,5]. Certain effector T cells differentiate into memory T cells (Tm cells), which possess an extended lifespan and are capable of eliciting a strong immune response upon subsequent exposure to the same pathogen [6]. This memory response underpins the effectiveness of vaccines, enabling the body to generate faster and stronger protective immunity against previously encountered pathogens.

Lactate production is a fundamental aspect of the metabolic processes in cancer cells, arising from heightened glycolytic activity that continues to occur in the presence of oxygen. This phenomenon is commonly known as the Warburg effect [7]. Lactate, long dismissed as a mere glycolytic byproduct, was once thought to be cellular waste in cancer cells [8]. However, with continued research, it has become evident that lactate plays a pivotal role in cellular metabolism and acts as a signaling molecule across various pathways [9]. In the TME, high lactate levels contribute to an acidic environment that can inhibit immune cell activity and facilitate tumor advancement, and lactate is a key mediator, linking the two processes of metabolic reprogramming and immunosuppression [10]. Lactate plays a complex role in immune suppression. Research has shown that it decreases the expression of critical immune molecules and receptors on T cells, thereby impairing their ability to recognize and attack tumor cells [11]. Moreover, lactate functions as a signaling molecule that recruits immunosuppressive cells, such as Tregs and myeloid-derived suppressor cells (MDSCs), additionally attenuating the anti-tumor immune response [12]. This intricate relationship between lactate metabolism and the TME highlights the multifaceted role of lactate in facilitating tumor progression, while also influencing the functionality of immune cells.

Lactylation, a recently identified PTM characterized by the incorporation of lactate into lysine residues on both histones and non-histone proteins, highlights the intricate role that lactate plays in the field of cancer biology [13,14]. Histone lactylation has been linked to the transcriptional regulation of genes governing glycolysis and immune responses, thereby establishing a direct link between metabolic shifts and epigenetic control [15]. Further studies have demonstrated that lactylation is abundant in immune cells and linked to multiple forms of cancer [16,17]. Lactic acid, a crucial signaling molecule in cellular regulation, induces lactylation and alters protein structure and function. Recent research has demonstrated that lactylation is an essential aspect of lactic acid function, significantly influencing both physiological and pathological processes, including the progression of tumors [16], immune regulation [15], inflammation [18], and fibrosis [19]. The immunosuppressive properties of the TME present a considerable obstacle to the efficacy of cancer immunotherapy. Within this environment, lactate and lactylation seem to play significant roles in regulating T cell activity, which are the principal effectors of the immune response to tumors. This review explores the critical roles of lactate and lactylation in tumor metabolism, the TME, as well as the bidirectional regulation of T cell immunity. It also addresses the potential for targeting lactate and lactylation in the development of novel cancer therapies, including integrated targeted treatments and immunotherapy.

## 2. T Cell Metabolism in the Tumor Microenvironment

**Figure 1:**
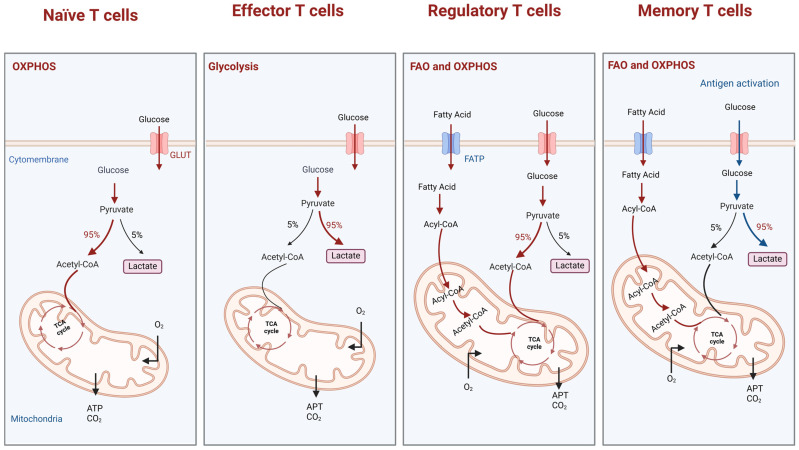
Metabolic pathways of different T cell subsets. Naïve and memory T cells primarily rely on OXPHOS and FAO to meet their energy demands. However, upon re-exposure to antigens, memory T cells switch to glycolysis. Activated effector T cells harness glycolysis to fulfill their rapid energy requirements. In contrast, regulatory T cells depend on OXPHOS to sustain their immunosuppressive functions. Abbreviations: OXPHOS: oxidative phosphorylation; FAO: fatty acid oxidation; FATP: fatty acid transport protein; TCA cycle: tricarboxylic acid cycle; ATP: adenosine triphosphate; GLUT: glucose transporter. Created with BioRender.com.

Modifications in T cell metabolic pathways within the TME significantly influence their functionality. In tumor cells, accelerated glucose metabolism depletes glucose in the TME, resulting in the excessive accumulation of lactate and a reduction in pH, creating an acidic environment that complicates immune cell function [20]. In their quiescent state, T cells predominantly rely on oxidative phosphorylation (OXPHOS) for the generation of sustained and stable levels of adenosine triphosphate, effectively meeting their low metabolic demands and ensuring proper functioning while remaining in an inactive state (Figure 1) [21]. However, following antigen stimulation, T cell metabolism undergoes a significant shift, heavily relying on glycolysis to meet their energy demands and produce lactate, even in aerobic conditions. This enhances the release of cytokines like IFN-γ, IL-2, and tumor necrosis factor-α (TNFα), which are essential for biosynthesis and the maintenance of their effector functions [22]. As glycolysis intensifies, lactate and hydrogen ions must be exported, a process mediated by monocarboxylate transporters (MCTs) [23]. However, the elevated extracellular lactate level disrupts this export, leading to the reabsorption of lactate and protons, which lowers the intracellular pH in T cells [24]. Lactate accumulation within T cells causes intracellular acidification, directly inhibiting glycolysis and ultimately reducing cytotoxic T lymphocyte (CTL) proliferation, cytotoxicity, and pro-inflammatory cytokine production [25,26].

These metabolic disruptions further impair anti-tumor immunity by interfering with key transcriptional regulatory mechanisms within the cell. The inhibition of glycolysis enables GAPDH to associate with the IFN-γ mRNA, preventing effective protein synthesis, thus reducing CTL cytokine secretion, and impairing their tumor-killing function [27]. Additionally, intracellular acidification affects the activity of nuclear factor of activated T cells (NFAT), a critical transcription factor. The acidic environment inhibits calcineurin, the phosphatase responsible for NFAT dephosphorylation, reducing its nuclear translocation and thereby limiting its activation of the IFN-γ gene [28].

Tregs, on the other hand, exhibit metabolic flexibility that supports their immunosuppressive function in various microenvironments (Figure 1). Unlike effector T cells, Tregs exhibit a greater metabolic reliance on fatty acid oxidation (FAO) and OXPHOS, especially in glucose-deficient environments such as the TME or inflamed tissues [29]. Through FAO, Tregs can maintain metabolic stability in low-glucose and oxidative stress environments, ensuring the proper execution of their immunosuppressive functions. Tregs rely on mitochondrial OXPHOS for energy metabolism, allowing them to survive and function under nutrient-limited and glucose-scarce conditions [29]. Unlike the glycolytic reliance noted in effector T cells, Tregs display notable metabolic flexibility. Tregs are less reliant on glycolysis and can instead metabolize lactate as a substrate, sustaining their proliferation and function through the tricarboxylic acid (TCA) cycle [30]. This metabolic adaptation not only supports Treg survival in nutrient-deprived conditions but also amplifies their immunosuppressive capacity, enabling them to regulate immune responses and suppress effector T cell activity within the TME.

Tm cells depend on lipid oxidation as a source of energy to sustain their fundamental metabolic processes (Figure 1). Upon reactivation, Tm cells rapidly switch to glycolysis, breaking down glucose to generate energy, meeting the demands of a swift immune response, and enhancing effector function [31]. However, the excessive enhancement of glycolysis can significantly impair the long-term survival of CD8^+^ Tm cells [32]. Overall, the metabolic changes in the TME profoundly affect the functions of T cell subtypes. Through glucose depletion, lactate accumulation, and pH reduction, the tumor environment not only weakens the anti-tumor efficacy of CTLs but also promotes the immunosuppressive function of Tregs, ultimately contributing to tumor immune evasion. These intricate metabolic mechanisms highlight the importance of modulating T cell metabolism in cancer therapies.

## 3. Characteristics of Lactylation

### 3.1. Histone Lactylation

**Figure 2:**
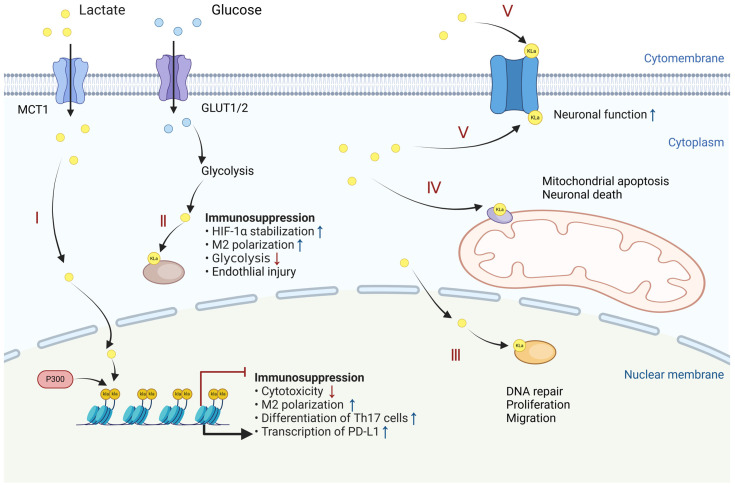
Lactylation occurs in various cellular compartments. Beyond nuclear histones that influence gene transcription, lactylation can also occur on non-histone proteins located in the membrane, cytoplasm, and nucleus, impacting immune function and energy metabolism. The diagram shows that lactylation occurs in different cellular compartments: I—histones; II—cytoplasmic proteins; III—nuclear proteins; IV—mitochondrial membrane proteins; and V—cell membrane proteins. Created with BioRender.com.

Histone lactylation (Kla) is a PTM that represents a significant advancement in understanding epigenetic regulation. Discovered by Zhao et al. in 2019, it refers to the modification of lysine residues on histone tails through the addition of a lactyl group, introducing a novel mechanism of chromatin remodeling [15]. This discovery has introduced a novel dimension to the dynamic landscape of histone modifications, joining the ranks of other well-established PTMs like acetylation, phosphorylation, and ubiquitination (Figure 2).

Histone modifications regulate chromatin architecture and activity through two principal mechanisms. The initial mechanism pertains to the direct alteration of the physical structure of chromatin, exemplified by processes such as histone acetylation and phosphorylation, which serve to neutralize the positive charge associated with histones. This weakening of the electrostatic interactions between histones and DNA results in a more relaxed chromatin state that facilitates the binding of transcription factors and other protein mechanisms essential for gene expression [33]. The second mechanism revolves around the recruitment of chromatin-associated factors, where specific histone modifications, such as H3K4me3 and H3K9me3, are recognized by protein structural motifs, including the PHD finger and Tudor motifs, which further regulate chromatin dynamics and gene transcription [34]. Notably, lysine residues in histones are exposed to the protein surface and are highly susceptible to emulsification, allowing this PTM to have an impact on chromatin structure and function [35]. This unique modification not only adds to the complexity of chromatin structure but has also been intensively studied in terms of functional regulation.

The functional implications of histone lactylation are profound, particularly regarding immune responses and cancer. Research revealed that histone H3K18 lactylation (H3K18la) is markedly upregulated during M1 macrophage activation, driven by enhanced glycolysis and lactate production. This modification promotes the transition of M1 to M2 macrophages, fostering a pro-tumor microenvironment. Inhibiting lactate production or H3K18la reverses this shift, underscoring lactylation’s pivotal role in immune regulation and tumor progression [15]. Similarly, in colon cancer, PCSK9-mediated lactate production induces histone lactylation, driving macrophage polarization and enhancing metastasis via the EMT and PI3K/AKT signaling pathways [17]. Furthermore, in inflammatory diseases such as colitis, lactate modulates histone modifications, thereby altering macrophage activity. Lactic acid treatment inhibits the activation of inflammatory pathways like NLRP3 while increasing H3K18la, suggesting a protective role in inflammation [18]. In tumor-infiltrating myeloid cells, histone lactylation promotes the expression of METTL3, leading to the m6A modification of the JAK1 mRNA and subsequent cytokine production, further supporting immunosuppressive functions [36]. These findings highlight the significant roles of histone lactylation in modulating immune responses and affecting the progression of diseases, especially in the contexts of cancer and inflammation.

### 3.2. Non-Histone Lactylation

The involvement of lactylation in the regulation of gene expression and cellular function has attracted considerable scholarly interest. This has led researchers to question whether lactylation is a prevalent PTM that also modifies non-histone proteins and what functional significance this might hold (Figure 2). One pivotal study utilized tandem mass spectrometry to identify a cyclic immonium ion of lactyllysine, which facilitated the reliable identification of lactylation in a wide variety of proteins present in the human proteome [13]. Glycolytic enzymes, notably aldolase A, undergo significant lactylation at lysine 147, a modification that appears to inhibit enzymatic activity. This observation supports a feedback mechanism whereby lactylation modulates glycolysis, suggesting its broader role in metabolic reprogramming [13]. This discovery indicates that lactylation is not confined to histones but is prevalent among various non-histone proteins, including those involved in glycolysis, underscoring the extensive impact of lactylation on cellular processes and highlighting the imperative for further investigation.

The lactylation of cytoplasmic proteins plays roles in various pathological processes, including immune suppression, inflammatory responses, angiogenesis, and tumor progression, highlighting its potential as a therapeutic target. The K62 lactylation of PKM2 enhances pyruvate kinase activity, maintaining glycolysis in M1 macrophages [37], while the lactylation of RIG-I inhibits the NF-κB signaling pathway, leading to immune suppression in M2 macrophages [38]. Lactylation of HIF-1α, Yin-Yang 1 (YY1), and Sox10 regulates angiogenesis and vascular proliferation through different mechanisms. HIF-1α promotes angiogenesis in prostate cancer via the KIAA1199 pathway [39], YY1 drives pathological retinal neovascularization under hypoxic conditions [40], and Sox10 induces vascular smooth muscle cell transdifferentiation and pyroptosis, contributing to vascular proliferation [41]. Additionally, the lactylation of HMGB1, mediated by p300/CBP, enhances its exosomal release, exacerbating sepsis progression [42].

Recent studies have emphasized the crucial role of lactylation in regulating nuclear proteins, which are central to maintaining genomic stability and promoting cancer progression. The MRN complex, composed of the nuclear proteins MRE11, RAD50, and NBS1, is essential for homologous recombination repair, a critical process for resolving DNA double-strand breaks. Lactylation has been shown to modulate the functions of these nuclear proteins, enhancing DNA repair mechanisms [43,44]. Additionally, lactylation of nucleolin (NCL), another key nuclear protein, has been linked to the progression of intrahepatic cholangiocarcinoma (iCCA), highlighting the broader impact of this modification on nuclear protein function in both DNA repair and tumor growth [45].

Cell membrane proteins play a crucial role in key physiological processes such as intracellular and extracellular signal transmission, energy metabolism, and apoptosis. A study identified 1003 lysine lactylation sites on 469 cortical proteins in a cerebral ischemia–reperfusion injury rat model, revealing significant lactylation modifications in mitochondrial membrane proteins that impact the Ca^2+^ signaling pathway, mitochondrial apoptosis, and neuronal death in acute ischemic stroke [46]. In neurobiology, the lactylation of cell membrane proteins, such as SNAP91, has been found to enhance synaptic resilience and neuronal function, especially under stress conditions, indicating a vital role for non-histone lactylation in the brain’s adaptive responses [47].

Collectively, these studies reveal that lactylation is a widespread and functionally diverse PTM that extends beyond histones to impact various cellular processes, including metabolism, inflammation, DNA repair, chemotherapy resistance, and the immune response. As research continues to uncover the full scope of non-histone lactylation, it holds promise as a key regulator in health and disease, potentially leading to novel therapeutic strategies for a range of conditions.

## 4. Lactate and Lactylation in T-Cell-Mediated Immune Stimulation and Suppression

### 4.1. Lactate in T-Cell-Mediated Immune Suppression

Lactate has been repeatedly shown to exert a direct influence on T-cell-mediated immune responses. It serves dual roles as both a metabolic substrate and a signaling molecule, modulating T cell activity in a manner that is dependent on the specific context.

Lactate exerts inhibitory effects on CD8^+^ T cells, affecting several critical aspects of their functionality. It has been shown to reduce the secretion of IFN-γ, TNF-α, and IL-2, which are essential for effective anti-tumor responses [25,48]. The activation of critical signaling pathways in T cells, including the NFAT and p38/JNK/c-Jun signaling pathways, is vital for cytokine production and cytotoxic activity [48,49]. Consequently, CD8^+^ T cells subjected to elevated levels of lactate exhibit reduced proliferation, cytotoxicity, and a greater susceptibility to activation-induced cell death, further diminishing their anti-tumor efficacy [50,51]. In addition to its direct metabolic effects, lactate promotes tumor immune evasion by modulating the expression of the ligands and receptors involved in immune recognition and response. For example, lactate can upregulate the expression of programmed death-ligand 1 (PD-L1) on tumor cells through its receptor GPR81, thereby inhibiting CD8^+^ T-cell-mediated cytotoxicity and promoting immune tolerance within the TME [52,53]. The multifaceted effects of lactate on CD8^+^ T cells not only diminish their functional capacity but also facilitate immune evasion by tumor cells.

Tregs are particularly adept at utilizing lactate as a metabolic substrate. Unlike effector T cells, Tregs display metabolic flexibility, enabling them to flourish in the high-lactate, low-glucose conditions of the TME. This metabolic adaptation supports their suppressive function, as lactate influx via MCT1 transporters is essential for sustaining Treg activity [30]. Lactate further enhances Treg stability by promoting OXPHOS and inhibiting glycolysis, a metabolic shift driven by high FOXP3 expression, which reprograms Treg metabolism to favor NAD+ oxidation [54]. In addition to facilitating the metabolic processes of Tregs, lactate also plays a crucial role in enhancing their suppressive capabilities. This enhancement occurs through the activation of critical signaling pathways, notably the PI3K/Akt/mTOR pathway, which leads to an upregulation of FOXP3 expression and strengthens the suppressive effects of Tregs on effector T cells [55,56]. Furthermore, lactate promotes the recruitment of Tregs to the TME by upregulating chemokines such as CXCL12 and CX3CL1, facilitating Treg infiltration, and contributing to the immunosuppressive environment [57,58]. Recent studies have elucidated the role of lactate in modulating the functions of CD4^+^ T cells within the tumor microenvironment and in the context of chronic inflammation. Specifically, exposure to cancer-associated fibroblasts results in a decrease in Th1 cell populations, which is mediated by the lactate-dependent degradation of T-box expressed in T cell (T-bet) by SIRT1. Concurrently, this exposure facilitates the differentiation of Tregs through the activation of the NF-κB and FoxP3 pathways [59]. Additionally, sodium lactate (NaL) skews CD4^+^ T cells towards a proinflammatory Th17 phenotype, which is accompanied by impaired T cell motility due to glycolysis disruption [24]. In chronic inflammation, lactate uptake via the SLC5A12 transporter reshapes CD4^+^ T cell effector functions, enhancing IL-17 production and fatty acid synthesis while retaining these cells in inflamed tissues. The blockade of SLC5A12 has shown potential in reducing disease severity in murine arthritis models [60]. The involvement of lactate in modulating the metabolism and functionality of Tregs, along with its influence on other subsets of CD4^+^ T cells, underscores its significant role in the immunosuppressive characteristics of the TME and in the context of chronic inflammation.

Natural killer T (NKT) cells, a distinct subset of T cells, exhibit a significant vulnerability to the immunosuppressive properties of lactate. Lactate induces apoptosis in NKT cells, reducing their numbers and impairing their ability to contribute to anti-tumor immunity [61]. Additionally, lactate inhibits the production of key cytokines in NKT cells by blocking mTOR signaling, a pathway crucial for their activation and function [62,63]. Furthermore, lactate has been implicated in increasing the expression of additional immunosuppressive molecules, such as Vam6 in invariant natural killer T cells, which correlates with impaired cytotoxic function [64]. Lactate further exacerbates the suppressive effect of TME on anti-tumor immunity by impairing NKT cell function through the induction of apoptosis. This impairment occurs through the induction of apoptosis, the inhibition of essential cytokine secretion, and the upregulation of immunosuppressive molecules.

Collectively, these findings emphasize the multifaceted role of lactate in promoting immune suppression within the TME, particularly through its effects on CD8^+^ T cells and Treg cells. Targeting lactate metabolism represents a potentially effective approach for mitigating immune suppression and improving the effectiveness of immunotherapies.

### 4.2. Lactylation in T-Cell-Mediated Immune Suppression

Lactylation has emerged as a key mechanism by which lactate exerts its immunosuppressive effects in the TME. Within T cells, lactylation influences the functions of essential transcription factors and regulatory proteins that play critical roles in maintaining immune balance (Table 1).

**Figure 3:**
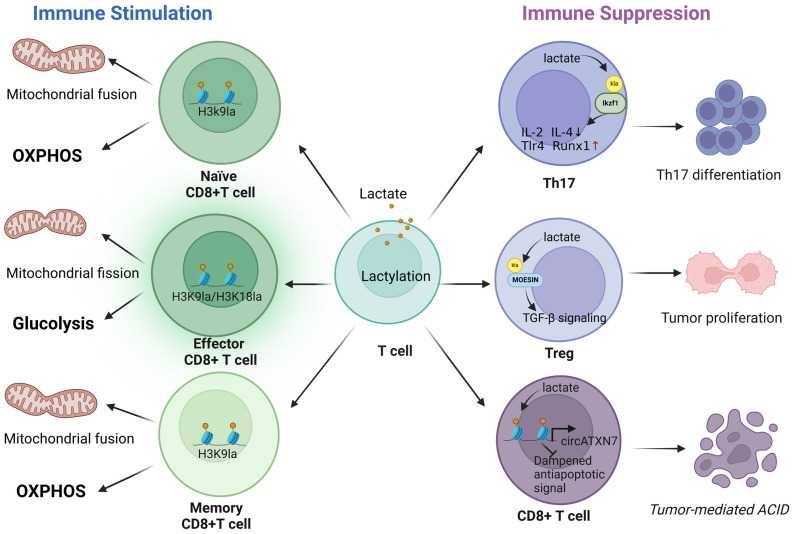
Lactylation in T-cell-mediated immunoactivation and immunosuppression. In distinct T cell subsets, on the one hand, histone lactylation can regulate the cell phenotype and function according to the metabolic state of T cells under certain conditions in the TME, and enhance the T-cell-mediated immune response. On the other hand, lactylation modulates the functions of different T cell subsets and promotes multiplex immunosuppression in the TME. Abbreviations: ACID: activation-induced cell death. Created with BioRender.com.

Histone lactylation is a critical mechanism through which lactate, a byproduct of glycolysis, influences gene expression. One of the critical roles of lactylation in immune suppression is its influence on Tregs, which are central to maintaining immune homeostasis (Figure 3). Lactate influences the production of Tregs through the lactylation of MSN (MOESIN), enhancing its binding to TGF-β receptors and SMAD3 signaling. This promotes OXPHOS in Tregs while inhibiting glycolysis, thereby boosting their suppressive function in the TME [70]. In malignant pleural effusion (MPE), lactate enhances the immunosuppressive properties of NKT-like cells by inducing the lactylation of FOXP3 [66]. Histone lactylation further amplifies the immunosuppressive environment by enhancing the transcription of ectonucleotidases CD39 and CD73, as well as the chemotactic receptor CCR8, which is a marker of tumor-infiltrating Tregs. This upregulation disrupts the Th17/Treg balance, favoring immune suppression and contributing to tumor progression [67]. Moreover, the lactylation of non-histone proteins can also influence T cell function. The lactylation of Ikaros family zinc finger 1 (IKZF1) at lysine 164, for example, enhances the differentiation of Th17 cells by upregulating the expression of Th17-associated genes, including Runx1, Tlr4, IL-2, and IL-4, further contributing to the suppression of anti-tumor immunity [68]. In KRAS-mutant tumors, lactate-induced histone lactylation activates the transcription of circATXN7, which interacts with the NF-κB p65 subunit, sequestering it in the cytoplasm and impairing NF-κB-mediated inflammatory responses. This mechanism promotes immune evasion and reduces the effectiveness of CTLs, which are crucial for anti-tumor immunity [50]. Moreover, the lactylation of RIG-I in macrophages leads to the suppressed recruitment of NF-κB to the Nlrp3 promoter, decreasing its transcription. This inhibition dampens the immunosuppressive functions of Tregs and the anti-tumor functions of CD8^+^ T cells, further contributing to an immunosuppressive TME [38]. In acute myeloid leukemia (AML), STAT5 is a transcription factor that promotes glycolysis and lactic acid accumulation, resulting in increased histone lactylation at the promoter region of PD-L1, a key immune checkpoint molecule. Histone lactylation enhances the transcription of PD-L1, leading to the inhibition of CD8^+^ T cell activation and facilitating immune escape in AML [69]. These findings highlight the pivotal role of lactylation in coordinating immune suppression in the TME, positioning it as a potential therapeutic target for interventions aimed at re-establishing effective anti-tumor immune responses.

### 4.3. Lactate in T-Cell-Mediated Immune Stimulation

As a metabolic substrate, lactate contributes to immune regulation that encompasses more than just immunosuppression. Lactate has been shown to enhance cytokine production in T cells following TCR activation. This effect relies on the availability of glycolytic intermediates and is particularly pronounced under metabolic stress conditions. For example, the inclusion of NaL during in vitro T cell activation markedly enhances the secretion of IFNγ, IL-2, and TNFα, thereby enhancing the effector functions of T cells [22]. Moreover, lactate serves as a preferred carbon source for CD8^+^ T cells during infection. In vitro studies demonstrate that CD8^+^ T cells preferentially rely on lactate rather than glucose for fueling the tricarboxylic acid cycle (TCA cycle), boosting their bioenergetic and biosynthetic capabilities. Blocking lactate-driven metabolism impairs both metabolic homeostasis and proliferative expansion, underscoring lactate’s critical role in sustaining T cell functions during immune responses [72]. Interestingly, lactate also modulates the epigenetic landscape of T cells. For instance, indole-3-lactic acid, a lactate derivative, enhances IL12a production in dendritic cells by promoting H3K27 acetylation at enhancer regions of the IL12a gene. This modification primes CD8^+^ T cell immunity against tumor growth, highlighting the complex interplay between lactate metabolism and T-cell-mediated immune stimulation [73]. One of the most intriguing aspects of lactate’s role in immune regulation is its promotion of CD8^+^ T cell stemness. This effect is mediated by the inhibition of histone deacetylase (HDAC) activity, which increases acetylation at the H3K27 site of the Tcf7 super-enhancer locus, upregulating Tcf7 expression and maintaining the stemness and proliferative potential of CD8^+^ T cells [74]. Additionally, lactate enhances the secretion of granzyme B and IFN-γ, as well as co-stimulatory markers like CD44, 4-1BB, and ICOS, thereby boosting the cytotoxic activity of CD8^+^ T cells [75]. This upregulation is linked to a coordinated increase in the expression of genes involved in the TCA cycle, reflecting a significant impact on T cell metabolism [75]. Furthermore, lactate enhances the phagocytosis and maturation of dendritic cells, which promote CD8^+^ T cell immune responses and inhibit tumor growth. Lactate also increases the number of interferon-gamma-expressing CD4^+^ and CD8^+^ T cells in the spleen and lymph nodes [76]. These studies indicate that lactate plays multiple roles in T-cell-mediated immune stimulation, including the promotion of cytokine production, regulation of epigenetic changes, and maintenance of T cell stemness and cytotoxicity. This underscores the complex and critical role of lactate in modulating T cell function.

### 4.4. Lactylation in T-Cell-Mediated Immune Stimulation

Lactate and lactylation are central regulators of T cell function, with significant effects on both immune suppression and activation. In the TME, these processes are critical for immune evasion and tumor progression, as they influence the activity of key immune cell populations (Figure 3).

H3K18la and H3K9la mark active promoters and enhancers, influencing the expression of key genes that determine the CD8^+^ T cell state. H3K9la is linked to mitochondrial fusion in naïve and memory CD8^+^ T cells, while H3K18la promotes mitochondrial fission in activated CD8^+^ T cells, which is crucial for their elevated glycolytic activity [22]. Interestingly, exogenous lactate fails to significantly impact H3K18la and H3K9la levels in activated CD8^+^ T cells, likely because the high levels of endogenous lactate produced during activation saturate these marks. However, in naïve and memory CD8^+^ T cells, exogenous lactate enhances histone lactylation, indicating a differential impact based on the metabolic profile of the T cell subsets [22]. The decrease in CD8^+^ T cell effector function after lactylation inhibition via LDHA inhibitors underscores the significance of these modifications in regulating T cell activity. Modulating histone lactylation has the potential to augment the anti-tumor capabilities of CD8^+^ T cells, thereby representing a novel approach for cancer immunotherapy [22]. These findings suggest that lactylation is a context-dependent regulator of T cell function with the potential to either suppress or stimulate immune responses, depending on the metabolic state of the T cells.

Lactate and lactylation are pivotal regulators of T cell function, with profound implications for both immune suppression and activation. Within the TME, these processes play crucial roles in immune evasion and tumor progression by modulating the activity of key immune cells. However, under certain conditions, lactate and lactylation can also enhance T-cell-mediated immune responses, offering new avenues for therapeutic intervention.

## 5. Lactate and Lactylation in T-Cell-Mediated Cancer Immunotherapy

### 5.1. Lactate in T-Cell-Mediated Cancer Immunotherapy

Checkpoint inhibitors have received approval for the treatment of cancer and have shown the ability to induce sustained responses in certain patients. Nevertheless, the phenomenon of resistance poses a considerable challenge to their effectiveness, and is primarily influenced by elements within the TME. The TME represents a complex ecosystem that facilitates tumor proliferation, survival, and evasion of the immune system. Among the various components that shape this microenvironment, lactate has been identified as a significant factor modulating the immune response, particularly in the context of T-cell-mediated cancer immunotherapy. Therefore, targeting lactate metabolism in conjunction with immune checkpoint blockade represents a promising approach to rejuvenate T cell functionality and improve the overall efficacy of immunotherapeutic strategies.

Lactate contributes to tumor immune evasion by adjusting the levels of immune checkpoint molecules. For example, in the TME characterized by elevated lactate levels, lactate promotes the expression of PD-L1 in lung cancer cells through the activation of the GPR81 receptor. This interaction initiates downstream signaling cascades that result in the inhibition of protein kinase A activity and the activation of the TAZ-TEAD complex. This cascade not only upregulates PD-L1 but also diminishes IFN-γ production, ultimately shielding tumor cells from cytotoxic T cell attacks [53]. Moreover, the lactate-mediated upregulation of programmed death receptor 1 (PD-1) in Tregs within highly glycolytic tumors further exacerbates immune suppression. In a low-glycemic environment, the expression of PD-1 in Tregs is enhanced by the production of excess lactic acid from glucose consumption by tumor cells, promoting NFAT1 translocation and Treg-mediated immune evasion. Blocking PD-1 in these contexts invigorates PD-1-expressing Tregs, leading to treatment failure [77]. A study found that lactate enhances USP39-mediated RNA splicing, promoting cytotoxic T lymphocyte-associated antigen-4 expression in a Foxp3-dependent way, which boosts Treg suppressive function in colorectal cancer patients [78]. These studies emphasize the important role of lactate in promoting immune evasion and providing a potential treatment target for enhancing the efficacy of checkpoint inhibitors.

#### 5.1.1. Targeting Lactate Production in Cancer Immunotherapy

**Figure 4:**
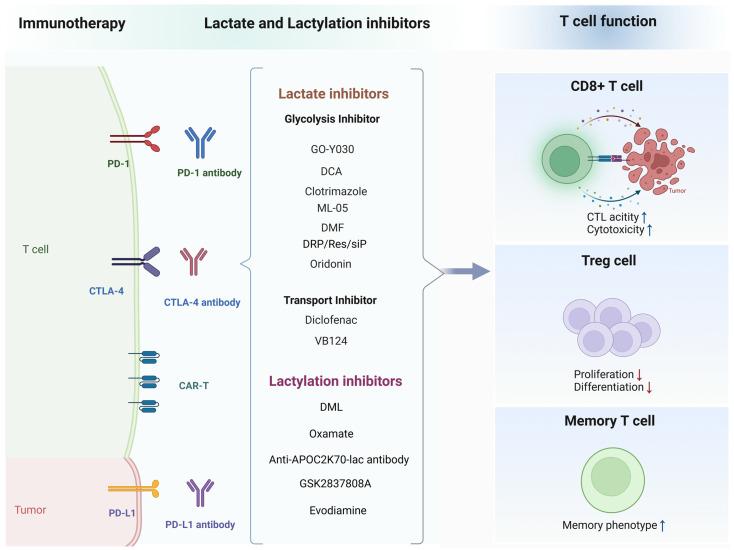
The combination of lactate and lactylation inhibitors with immunotherapeutic agents. Combination therapy targeting the TME promotes CD8^+^ T cell infiltration and enhances their cytotoxicity, thereby strengthening targeted tumor suppression. Abbreviations: CAR-T: chimeric antigen receptor T cells; CTL: cytotoxic T lymphocyte. DCA, dichloroacetate; DMF, dimethyl fumarate; DML, demethylzeylasteral. Created with BioRender.com.

Considering the central functions of lactate in immune suppression and tumor immune evasion, targeting lactate dehydrogenase A (LDHA) represents a promising strategy for enhancing the efficacy of cancer immunotherapy (Figure 4). LDHA is a crucial enzyme involved in the production of lactate, facilitating the conversion of pyruvate to lactate in anaerobic environments. In tumor cells, LDHA facilitates lactate production due to their reliance on enhanced glycolysis for energy, supporting rapid tumor growth and division [79]; pyruvate generated from glutamine metabolism is also converted into lactate via LDHA [80]. Inhibiting LDH activity has been shown to effectively reduce lactate production and improve the tumor microenvironment. In addition to extensively studied LDH inhibitors such as oxamic acid (oxamate) [81], FX-11 [82], quinoline-3-sulfonamide [83] and galloflavin [84], polyphenolic compounds such as quercetin and resveratrol exhibit significant LDH inhibitory effects [85]. Quercetin not only directly inhibits LDH activity but also suppresses lactate production and cancer cell growth by downregulating LDH-related gene expression [85]. A newly developed LDHA inhibitor, ML-05, effectively decreases lactate synthesis, leading to both tumor growth inhibition and enhanced antitumor immune responses of CD8^+^ T cells. Furthermore, when ML-05 is combined with other immunotherapies, such as anti-PD-1 antibodies, it significantly amplifies anti-tumor efficacy, offering a promising strategy for cancer treatment [86]. RNA interference technology also demonstrates strong potential as an LDH inhibitor, offering promise as an important tool in future disease treatment by interfering with the expression of LDH-related genes [87].

#### 5.1.2. Targeting Glycolysis in Cancer Immunotherapy

The inhibition of lactate production through glycolysis can significantly impact T cell infiltration and function (Figure 4). For instance, modifying metabolic pathways to reduce lactate production can significantly affect the infiltration and functionality of T cells within tissues. The application of a dual-responsive mPEG-PLA-PHis-ss-PEI polypolymer (DRP/Res/siP) for the purpose of downregulating glycolytic pathways and upregulating mitochondrial OXPHOS in tumor cells resulted in a decrease in lactate production, an enhancement of the infiltration of CD8^+^ and CD4^+^ T lymphocytes, and improved anti-tumor efficacy when utilized in conjunction with PD-L1 silencing [88]. In bladder cancer, oridonin inhibits lactate-induced PD-L1 expression and amplifies the cytotoxicity of CD8^+^ T cells when paired with a PD-L1 inhibitor [89]. Inhibiting the IL-8/CXCR2 pathway in gastric cancer lowers PD-L1 expression and lactate production, enhancing the effectiveness of anti-PD-1 immunotherapy [90]. Further research showed that tumor-derived L-lactate enhances Treg suppressive function. The curcumin analog GO-Y030 reduced L-lactate production by inducing metabolic changes and decreasing mTOR-S6 axis activity, which is key for Treg function. GO-Y030 also altered the metabolism of cultured CD4^+^ T cells exposed to TGF-β and IL-6, selectively inhibiting IL-10 production in Th17 cells without affecting Th17 differentiation [91]. Hexokinase 2 (HK2), a member of the hexokinase family, acts as a key initiator of the glycolytic pathway. Clotrimazole, by specifically inhibiting HK2, regulates lactic acid production and promotes the maturation of dendritic cells. This modulation enhances T cell activation and facilitates intra-tumoral immune infiltration, thereby significantly improving the therapeutic efficacy of anti-PD-1 treatment [92]. Glyceraldehyde-3-phosphate dehydrogenase (GAPDH) is a key enzyme in the glycolysis pathway, catalyzing a reaction accompanied by the production of nicotinamide adenine dinucleotide (NADH). Beyond its canonical role in glycolysis, NADH generated by GAPDH is pivotal for maintaining intracellular redox homeostasis and modulating reactive oxygen species levels [93]. Notably, the inhibition of GAPDH by compounds such as dimethyl fumarate, which are traditionally used for autoimmune disorders, has demonstrated efficacy in impairing both aerobic glycolysis and OXPHOS within tumor cells [94]. Pyruvate dehydrogenase kinase 1 (PDHK1) serves as a critical enzyme within the glycolytic pathway. Targeting PDHK1 offers a promising strategy to enhance immune cell function [95]. Inhibitors such as dichloroacetate (DCA) have been shown to downregulate PDHK1 expression, promoting the secretion of key cytokines like granzyme B, IFN-γ, and TNF-α. This mechanism has demonstrated the potential to slow cancer progression and enhance the effectiveness of immunotherapy [96].

#### 5.1.3. Targeting Lactate Transport in Cancer Immunotherapy

Inhibiting lactate production and transport has emerged as a strategy to enhance the efficacy of cancer immunotherapy (Figure 4). MCTs are key proteins involved in lactate transport. Among them, MCT1 has a high affinity for lactate and primarily facilitates the uptake of lactate into the cell, while MCT4, which is upregulated under hypoxic conditions, has a lower affinity and primarily mediates lactate efflux from the cell. For instance, inhibiting MCTs reduces lactate efflux, thereby improving the immune response by increasing CD8^+^ T cell infiltration and cytotoxicity. Combining lactate metabolism inhibitors with immune checkpoint inhibitors (ICIs) has shown promising results in preclinical models, suggesting that this approach could enhance the effectiveness of current immunotherapies [97]. This has also been demonstrated in Tregs [30]. For instance, diclofenac, an inhibitor of MCT1 and MCT4, reprograms tumor glycolysis, reverses tumor acidification, and enhances the effectiveness of checkpoint therapy by promoting immune cell-driven anti-tumor responses [98]. Similarly, the m6A demethylase Alkbh5 modulates Mct4/Slc16a3 expression and lactate levels in the TME, enhancing the efficacy of cancer immunotherapy by altering the composition of tumor-infiltrating Tregs and MDSCs [99]. In hepatocellular carcinoma (HCC), the inhibition of MCT4, either genetically or pharmacologically with VB124, has been shown to suppress tumor growth by enhancing CD8^+^ T cell infiltration and cytotoxicity. This effect is attributed to the reduced acidification of the TME and increased secretion of chemokines like CXCL9 and CXCL10 mediated by reactive oxygen species/NF-κB signaling. Notably, combining MCT4 inhibition with anti–PD-1 immunotherapy significantly improves therapeutic outcomes in patients with HCC [100]. The use of mitochondrial pyruvate carrier (MPC) inhibitors during the manufacturing of chimeric antigen receptor (CAR) T cells has also demonstrated promising results. MPC inhibition promotes the development of a memory phenotype in chimeric antigen receptor T (CAR-T) cells, which is associated with sustained anti-tumor activity, underscoring the importance of metabolic modulation in enhancing the efficacy of CAR-T cell therapy [101]. The MCT1 inhibitor BAY-8002 effectively blocks the bidirectional transport of lactate and demonstrates significant antiproliferative activity against specific types of cancer cells, particularly diffuse large B cell lymphoma and some solid tumor models [102]. Similarly, the use of lithium carbonate has demonstrated potential in rescuing CD8^+^ T cells from lactate-induced immunosuppression by blocking lysosomal acidification and facilitating the use of lactate as an energy source [103].

#### 5.1.4. Lactate-Regulating Nanomedicine Systems

Recent advancements in the integration of nanotechnology with lactate inhibitors have demonstrated substantial potential in cancer therapy. Nanocarrier systems overcome the limitations of traditional inhibitors, such as poor stability and limited specificity, by enhancing drug targeting and retention [104,105]. Recent studies have demonstrated that 2-deoxy-D-glucose (2-DG)-encapsulated poly(lactic-co-glycolic acid) (PLGA) nanoparticles (2DG-PLGA-NPs) effectively combine lactate inhibition with nanotechnology, significantly reducing lactate production, enhancing T cell functionality, and suppressing tumor proliferation [106]. Moreover, the pH-responsive AZD3965 nanomedicine rapidly releases its drug in acidic environments and, when combined with anti-PD-1 therapy, significantly enhances survival rates and reduces the drug dosage by over 200-fold compared to oral AZD3965 [107].

### 5.2. Lactylation in T-Cell-Mediated Cancer Immunotherapy

Lactylation, a recently identified PTM, has emerged as a key regulator of immune responses within the TME. Emerging evidence highlights the diverse roles of lactylation in modulating cancer immunotherapy efficacy, particularly in relation to ICIs such as anti-PD-1 therapy.

Recent studies have demonstrated that lactylation levels in Treg cells play a crucial role in determining modulating the efficacy of anti-PD-1 therapy in HCC patients. These data indicated that patients who responded to anti-PD-1 therapy exhibited lower levels reduced MOESIN lactylation in Treg cells compared to non-responders. This suggests that lactylation may enhance the immunosuppressive functions of Treg cells, thereby hindering the anti-tumor immune response. Moreover, the combination of anti-PD-1 therapy with lactate dehydrogenase inhibitors, which suppress lactate production and consequently lactylation, resulted in enhanced anti-tumor activity compared with anti-PD-1 therapy alone [70]. Oxamate, a lactate generation inhibitor, has shown promise in enhancing the efficacy of CAR-T therapy against glioblastoma by altering immune molecule phenotypes and increasing Treg cell infiltration. Lactate accumulation upregulated CD39, CD73, and CCR8 expression through histone H3K18 lactylation, suggesting that targeting lactate metabolism could reprogram glucose metabolism and alleviate immunosuppression in the tumor microenvironment [67]. Recent studies have confirmed that lactylation, particularly H3K18la, plays a critical role in T-cell-mediated cancer immunotherapy by promoting immune evasion through the POM121/MYC/PD-L1 axis in non-small cell lung cancer (NSCLC). Targeting this modification with glycolysis inhibitors (2-DG and oxamate) combined with anti-PD-1 antibodies can significantly enhance CD8^+^ T cell function [108]. In another NSCLC study, lactate enhanced the lactylation of APOC2 at lysine 70, stabilizing the protein and promoting the release of free fatty acids. This process resulted in the accumulation of Tregs, contributing to immunotherapy resistance and metastasis. The development of an anti-APOC2K70-lac antibody has shown promise in sensitizing tumors to anti-PD-1 therapy, suggesting a potential new approach in combination therapy for NSCLC [38]. Prostate cancer (PCa) represents another example where lactylation plays a critical role. Lactate-induced upregulation of HIF-1α and PD-L1, coupled with the suppression of Sema3A, facilitates tumor progression. However, evodiamine, a natural alkaloid, has been shown to reverse these effects by blocking lactate-induced histone lactylation, enhancing Sema3A expression, and inhibiting angiogenesis. These findings highlight evodiamine as a promising candidate for PCa therapy, offering a metabolic–epigenetic approach to overcome resistance [71]. In MPE, FOXP3+ NKT-like cells promote histone lactylation and maintain their immunosuppressive function through the high expression of MCT1 and lactate dehydrogenase B. Studies have shown that the MCT1 inhibitor 7ACC2 significantly reduces FOXP3 expression in NKT-like cells and decreases histone lactylation levels [66].

Several compounds have been shown to effectively inhibit lactylation, thereby influencing tumor initiation and progression. For instance, the demethylzeylasteral (DML) inhibits lactylation at H3K9la and H3K56la, suppressing tumorigenesis in liver cancer stem cells [109]. The royal jelly acid exerts potent anti-tumor effects by modulating the glycolytic pathway and regulating lactylation at the H3K9la and H3K14la histone sites [110]. Glutamine reduces lactate accumulation and lactylation in intervertebral disc degeneration models, enhancing autophagy and matrix synthesis [111]. Metformin diminishes histone lactylation, reducing oxidative stress and inflammatory responses [112].

Lactate and lactylation modifications inhibit T cell immune activity, resulting in a diminished tumoricidal capacity. In lactic acid-rich environments or under the influence of lactylation, tumor cells display increased resistance to cytotoxic effects. As a pivotal tumor suppressor, p53 functionality is compromised by lactic acid originating from tumors, specifically through lactylation of the K120 and K139 residues. This modification markedly reduces the DNA binding capacity and transcriptional activity of p53, consequently promoting tumor advancement [113]. Consequently, targeting lactate and its lactylation represents a promising therapeutic strategy. Targeting lactate metabolism, by disrupting its transport and production, has gained recognition as a potential therapeutic approach in cancer treatment. Despite advances in the development of metabolic inhibitors, clinical translation has remained challenging (Table 2). Recently, several agents have shown considerable promise in preclinical studies, with some advancing to clinical trials, although the outcomes have been mixed. For instance, 2-DG, which exhibited limited efficacy as a monotherapy, is now being explored in combination therapy trials (NCT00096707). Therefore, the discovery of lactylation and its central role in immune regulation represents a new avenue for cancer immunotherapy. The application of lactate or lactylation inhibitors enhances T cell immune activity, significantly improving their ability to effectively target and eliminate tumor cells, while concurrently decreasing tumor cell resistance and facilitating their destruction. Combining ICIs with agents that target lactate metabolism and lactylation may offer a powerful strategy for enhancing anti-tumor immunity.

## 6. Conclusions and Future Perspectives

Lactate and lactylation, key regulatory factors, play pivotal roles in various cellular activities, particularly in modulating T cell functions. A deeper understanding of lactate and its lactylation mechanisms, especially their influence within the TME, provides valuable insights into cancer progression and immune evasion. This review delves into the pivotal roles of lactate and lactylation in the regulation of tumor immunity, particularly within the context of dual interactions involving T cell immunity. The intricate relationship between lactate metabolism and immune regulation unveils novel therapeutic strategies aimed at disrupting lactate production or targeting lactylation, thereby enhancing the efficacy of immunotherapy and effectively countering tumor immune evasion. This perspective allows researchers to explore more precise intervention strategies, ultimately advancing clinical outcomes in cancer treatment.

However, the complexity of lactate and lactylation in T cell regulation poses several challenges. Firstly, lactate and lactylation exert dual roles in both immune suppression and immune activation, forming a complex regulatory network. Lactate not only directly inhibits T cell function but also modifies critical immune-related proteins through lactylation, further promoting tumor immune evasion. Under certain conditions, lactate and lactylation may enhance immune responses by facilitating the activation and stimulation of T cells. Understanding the balance of this dual regulation requires further investigation, particularly how lactylation enzymes—such as the “writers” p300/CBP [42], the “erasers” HDAC1-3 [152], and the recently identified “readers” Brg1 [153]—coordinate lactate’s and lactylation’s synergistic effects in different cellular environments.

Secondly, targeting lactate and lactylation presents several challenges, primarily due to the widespread presence of enzymes within the lactate metabolism pathway, which may lead to systemic toxicity. Current research is beginning to address these issues, with advancements in nanotechnology positioning nanoparticles as promising delivery vehicles capable of simultaneously and precisely transporting multiple therapeutic agents to tumor tissues through distinct mechanisms [106]. Furthermore, considering the various PTMs that many proteins undergo [154], the interactions between lactylation and other modifications—particularly acetylation—underscore the urgent need for further exploration of PTM crosstalk [42,155]. This intricate PTM network paves the way for the development of more targeted and effective therapeutic strategies, highlighting the potential for innovative approaches in cancer treatment.

Thirdly, though lactylation has primarily been studied in histones, its role in modifying non-histone proteins, such as membrane proteins, remains largely unexplored. Membrane proteins are critical mediators of signal transduction, recognition, and adhesion between the cell and its external environment, particularly within the immune system. Expanding research in this area could provide novel insights into the mechanisms of immune evasion. Recent studies reveal that the lactate-induced lactylation of NBS1 [44] and MRE11 [43] plays a crucial role in homology-directed repair of DNA, a mechanism that enhances the resistance of tumor cells to chemotherapy. Lactylation has raised intriguing questions about whether similar modifications could occur on RNA and DNA, potentially contributing to tumor development and tumor therapy resistance. Addressing these hypotheses underscores the critical role of advancements in mass spectrometry and high-throughput screening technologies. Such progress is pivotal for comprehensively defining the scope of lactylation and elucidating its role in immune regulation. A deeper understanding of these modifications may reveal novel targets and mechanisms in cancer immunotherapy.

Addressing these challenges will help identify the extensive scope of lactylation in T cells and reveal its role in tumor immune evasion. By advancing our understanding of lactate and lactylation in tumor immunity, more effective targeted therapies can be developed to enhance immunotherapy outcomes and overcome the challenges posed by tumor resistance.

## Figures and Tables

**Table 1:** The function of the lactylation modification in different T cell types and sites.

Modification Type	Disease	Site	Immune Cell	Function	Ref.
Histone lactylation	KRAS-mutant cancers	Histone	CD8^+^ T cell	Enhanced the sensitivity of CTLs to AICD	[50]
Histone lactylation	Intestinal inflammation	H3K18	Th17	Reduced IL17A production and upregulated Foxp3 expression through ROS-driven IL-2 secretion	[65]
Histone lactylation	Malignant pleural effusion	Histone	NKT	Maintained the immunosuppressive function	[66]
Histone lactylation	Glioblastoma multiforme	H3K18	T cell	Inhibited CD39 promoter activity and reduced CCR8 levels by blocking CCR8 binding to the macrophage-secreted ligands CCL1 and CCL18	[67]
Cytoplasmic protein lactylation	Autoimmune uveitis	Ikzf1 K164	TH17	Regulated TH17 differentiation by activating the transcription of Runx1 and Tlr4 and inhibiting the transcription of IL-2 and IL4	[68]
Cytoplasmic protein lactylation	Acute myeloid leukemia	H4K5	T cell	Enhanced PD-L1 transcription	[69]
Cytoplasmic protein lactylation	Hepatocellular carcinoma	MOESIN	Treg	Enhanced the immunosuppressive functions of Treg cells	[70]
Histone lactylation	Prostate cancer	H3K18	T cell	Increased PD-L1 expression and inhibited Sema3A transcription	[71]

Abbreviations: CTLs, cytotoxic T lymphocytes; AICD: activation-induced cell death; IL17A, interleukin-17A; Foxp3: forkhead box protein P3; ROS: reactive oxygen species; CCR8: CC chemokine receptor 8; CCL1: chemokine (C-C motif) ligand 1; TH17, T helper cell 17; Runx1, runt-related transcription factor 1; Tlr4, Toll-like receptor 4; PD-L1, programmed death ligand 1; IKZF1, Ikaros family zinc finger 1.

**Table 2:** Drugs targeting lactate metabolism and related clinical studies.

Target	Drugs	Stage	Tumor Type	Trial No.	Ref.
GLUTs	Phloretin	Pre-clinical	Colorectal cancer		[114]
	Fasentin	Pre-clinical	Neuroendocrine tumor, lung cancer		[115,116]
	STF-31	Pre-clinical	Melanoma, pancreatic cancer, breast cancer, glioblastoma		[117]
	WZB117	Pre-clinical	Glioblastoma, head and neck cancer		[117]
	Cytochalasin B	Pre-clinical	Lung cancer		[118]
	BAY876	Pre-clinical	Head and neck cancer, glioblastoma, breast cancer		[119,120]
HK	2-DG	Phase II	Hepatocellular carcinoma, breast cancer	NCT00096707	[121,122]
	Lonidamine	Phase II	Breast cancer, glioblastoma	NCT00435448	[123]
	Genistein-27	Pre-clinical	Breast cancer, colorectal cancer		[124]
	Benserazide	Pre-clinical	Colorectal cancer		[125]
	Resveratrol	Pre-clinical	Hepatocellular carcinoma, renal carcinoma		[126]
	Astragalin	Pre-clinical	Hepatocellular carcinoma		[127]
	Chrysin	Pre-clinical	Hepatocellular carcinoma		[128]
	3-BrPA	Pre-clinical	Breast cancer, lung cancer, pancreatic cancer, hematopoietic cancer		[129]
	WP1122	Phase I	Glioblastoma	NCT05195723	[130]
MCTs	α-CHCA	Pre-clinical	Glioblastoma, breast cancer		[131]
	BAY-8002	Pre-clinical	Hematopoietic cancer, breast cancer,		[102,132]
	VB124	Pre-clinical	Hepatocellular carcinoma		[100]
	AR-C155858	Pre-clinical	Colorectal cancer		[133]
	7ACC2	Pre-clinical	Breast cancer, oral squamous cancer, pancreatic cancer		[134,135]
	AZD3965	Phase I	Advanced cancer	NCT01791595	[136,137]
	Quercetin	Pre-clinical	Colorectal cancer		[138]
	SR13800	Pre-clinical	Neuroblastoma, lymphoma, breast cancer		[139]
	Syrosingopine	Pre-clinical	Hematopoietic cancer, lung cancer		[140]
	Fluvastatin	Phase I	Glioblastoma	NCT02115074	[141]
	Diclofenac	Phase Ⅳ	Basal cell carcinoma	NCT01935531	[98]
LDHA	FX-11	Pre-clinical	Pancreatic cancer		[82,142]
	Quinoline-3-sulfonamide	Pre-clinical	Lung cancer		[83]
	Oxamate	Pre-clinical	Cervical cancer, hepatocellular carcinoma		[81,143]
	Galloflavin	Pre-clinical	Breast cancer, hepatocellular carcinoma		[84]
	AT-101	Phase I	Prostate cancer	NCT00390403	[144]
	Thiazolidine-2,4-dione derivatives	Pre-clinical	Colorectal cancer, lung cancer, melanoma		[145]
	NHI-1	Pre-clinical	Ovarian cancer, pancreatic, colorectal cancer, mesothelioma		[146]
PFKFB3	3PO	Pre-clinical	Breast cancer, melanoma		[147]
GADH	Koningic acid	Pre-clinical	Hepatocellular carcinoma, pancreatic cancer		[148]
PGK	NG52	Pre-clinical	Autoimmune myocarditis		[149]
	GQQ-792	Pre-clinical	Glioblastoma		[150]
PDHKs	DCA	Phase I	Bladder cancer, hepatocellular carcinoma, breast cancer, prostate cancer	NCT00566410	[96,151]

Abbreviations: GLUTs, glucose transporters; HK, hexokinase; MCTs, monocarboxylate transporters; LDHA, lactate dehydrogenase A; PDHKs, pyruvate dehydrogenase kinases; GADH, glyceraldehyde-3-phosphate dehydrogenase; 2-DG, 2-deoxy-D-glucose; α-CHCA, α-cyano-4-hydroxycinnamic acid; 3PO, 3-(3-pyridinyl)-1-(4-pyridinyl)-2-propen-1-one; DCA, dichloroacetate; 3-BrPA, 3-bromopyruvate.

## Data Availability

Not applicable.
